# Supplementary material for: Fault rock heterogeneity can produce fault weakness and reduce fault stability
Source: Nat Commun. 2022 Jan 17;13:326. doi: 10.1038/s41467-022-27998-2 (PMC8763890; doi:10.1038/s41467-022-27998-2)
Supplement: Supplementary file 1 — Supplementary Material [file 41467_2022_27998_MOESM1_ESM.pdf]

**Fault rock heterogeneity can produce fault weakness and reduce fault stability: Supplementary material**

John D. Bedford[1*], Daniel R. Faulkner[1] & Nadia Lapusta[2,3]

[1]*Rock Deformation Laboratory, Department of Earth, Ocean and Ecological Sciences, University of Liverpool*

[2]*Department of Mechanical and Civil Engineering, Division of Engineering and Applied Science, California Institute of Technology*

[3]*Seismological Laboratory, Division of Geological and Planetary Sciences, California Institute of Technology*

*Corresponding author: jbedford@liverpool.ac.uk*

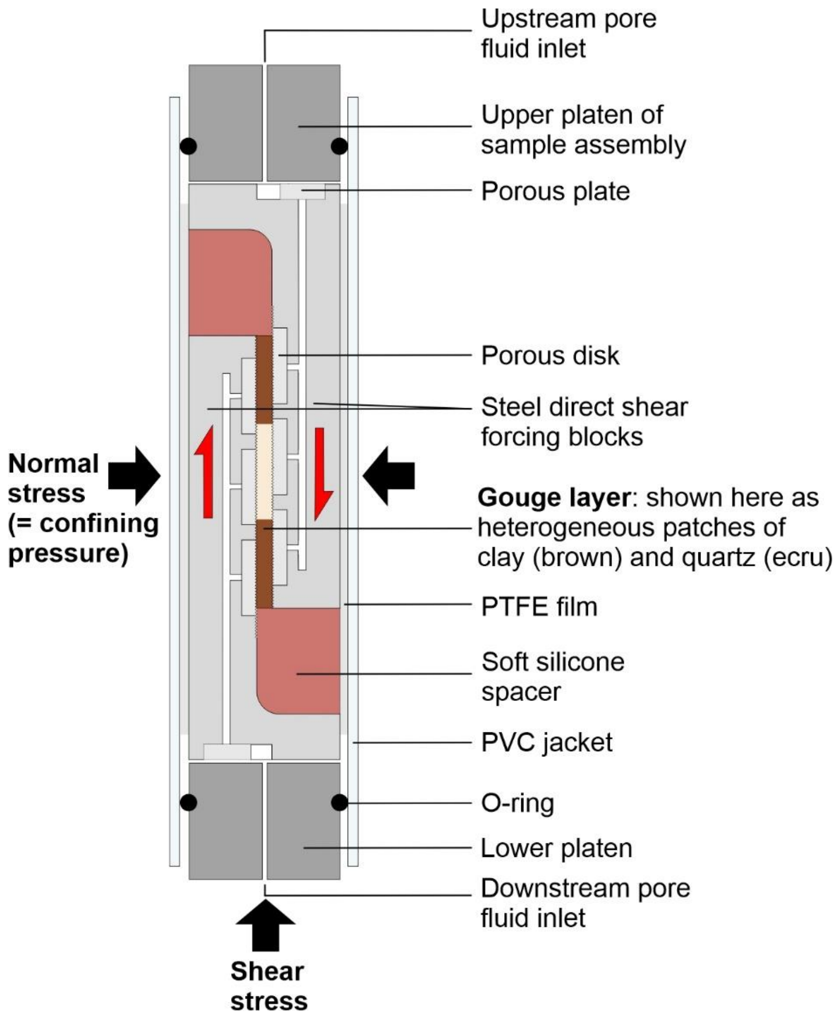


**Supplementary Figure 1| Schematic diagram of the direct shear arrangement used in the experiments.** The gouge layer is comprised of either heterogeneous patches of quartz and clay (as shown in this figure) or as a homogeneous quartz-clay mixture. The diameter of the platens is 20 mm and the length of the fault gouge layer is 50 mm.

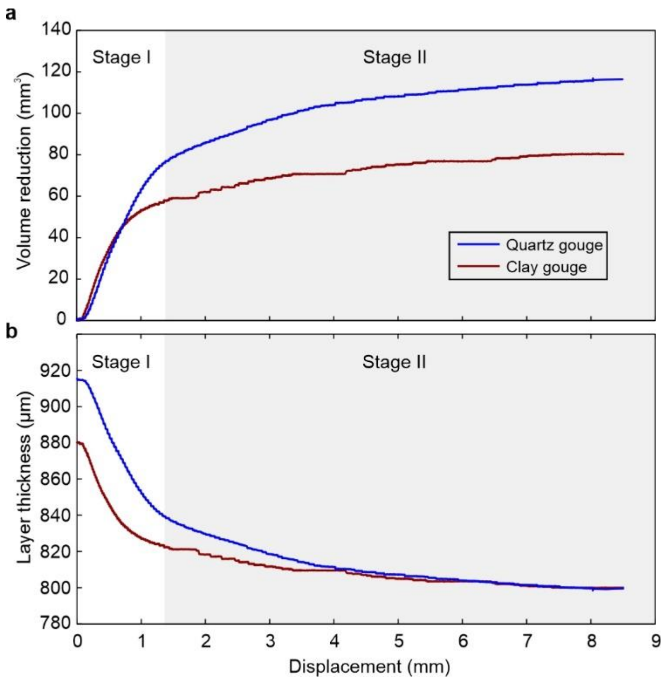


**Supplementary Figure 2| Pore volume and layer thickness data for the endmember quartz and clay gouges during shearing. a,** Pore volume reduction and **b,** layer thickness evolution of the gouge layers as they compact during an experiment. At the end of the experiment, the layer thicknesses were measured using a micrometer to be almost identical. The thickness evolution during the experiment was then back-calculated using the pore volume data, assuming the sliding area remains constant and that all volumetric strain is accommodated by a change in layer thickness[1] (see Methods in main article). The compaction evolution can be separated into two regimes. Stage (I): Loading of the gouge layers. Stage (II): Frictional shearing of the gouge layers, where the quartz gouge experiences a greater amount of shear enhanced compaction than the clay. While the endmember layer thicknesses do not differ much in the post-yield frictional shear regime, there is a difference of about 20 µm there. This difference can account for a significant fraction of the progressive weakening observed in the friction data of the heterogeneous faults (Fig. 1c) as discussed in Supplementary Note 1 below. Note that the reason the gouges do not have the same layer thicknesses at the start of the experiment is because the clay gouge compacts more than the quartz during initial pressurization to the starting conditions, as has been shown in previous work on clay-quartz gouges[2].

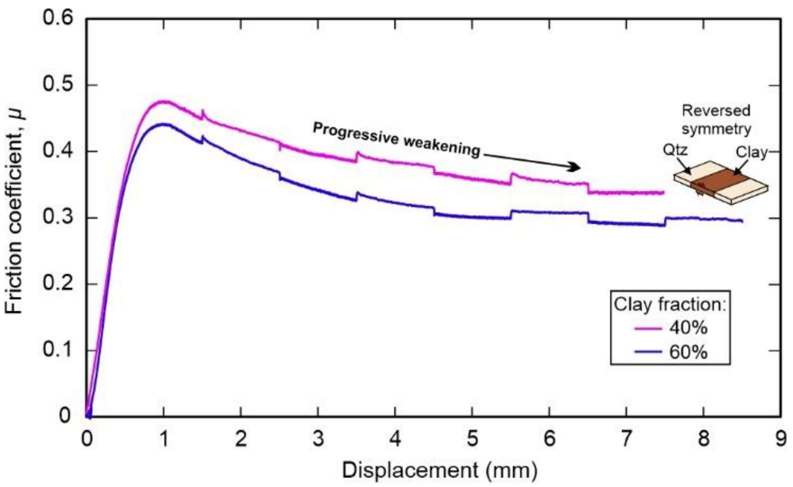


**Supplementary Figure 3| Evolution of the coefficient of friction (*μ*) with displacement for heterogeneous faults consisting of central clay patch bound by two quartz patches.** This data is from heterogeneous faults with opposite symmetry gouge layers to those presented in Fig. 1c of the main article. These heterogeneous faults exhibit similar weakening to those in Fig. 1c, showing that the arrangement of the different gouge patches in the layer does not dictate the overall frictional response and that progressive weakening is ubiquitous for all heterogeneous faults. Although similar weakening is observed, we note that some of the (*a* - *b*) values from the reversed symmetry faults are slightly higher than for the heterogeneous faults comprised of a central quartz patch (Supplementary Table 1). We have no explanation for this difference; although it could be due to (*a* - *b*) data from friction experiments generally being quite scattered, when compared to coefficient of friction data, and thus caution should be taken when directly comparing individual velocity steps.

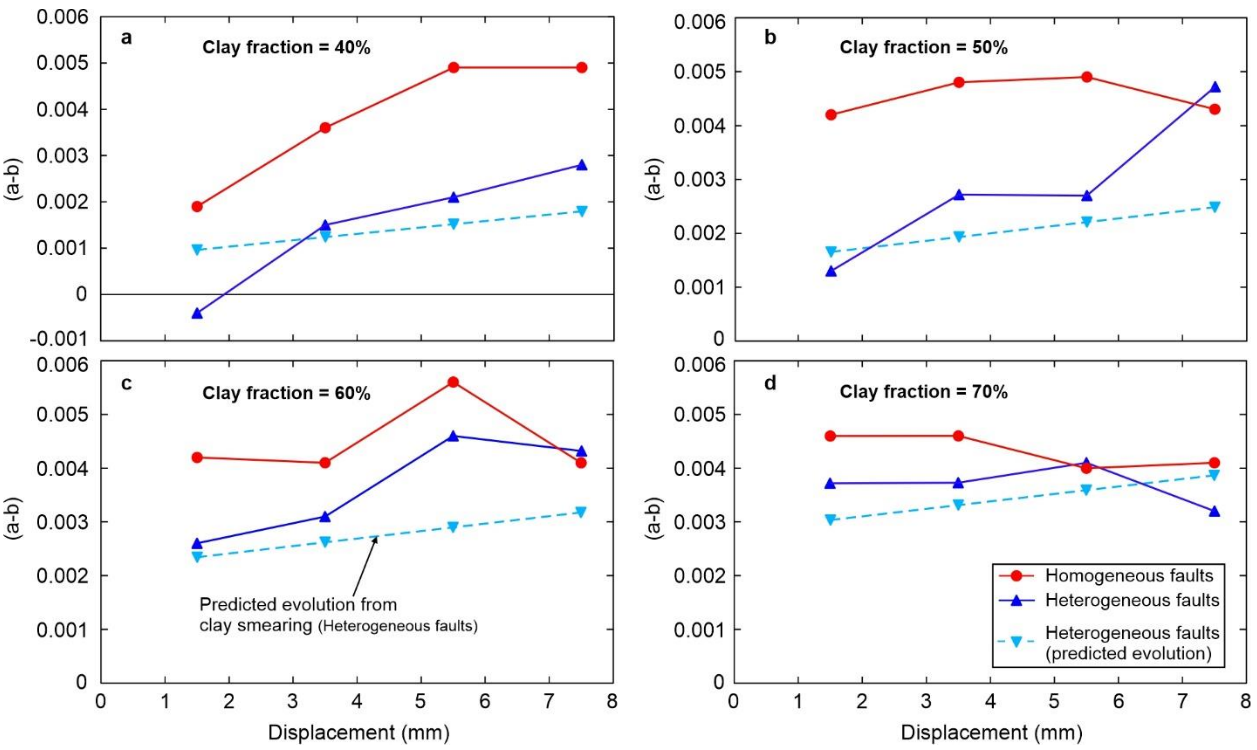


**Supplementary Figure 4| Predicted (*a* - *b*) evolution with displacement from clay smearing in the heterogeneous faults.** Also shown are the obtained (*a* - *b*) values from both heterogeneous and homogeneous fault experiments for clay fractions of **a.** 40%, **b.** 50%, **c.** 60% and **d.** 70%. The predicted (*a* - *b*) evolution is calculated using the arithmetic mean of the (*a* - *b*) values for the endmember quartz and clay gouges (from 1.5 mm displacement in Supplementary Table 1) and by assuming the length of the clay patch increases by the amount of displacement on the fault as the clay is smeared along localized Y-shear planes – as was done for coefficient of friction in Fig. 2c of the main article. The (*a* - *b*) data are more scattered than the friction data in Fig. 2c, so caution should be taken when interpreting the results; however, the data show that clay smearing generally underpredicts the (*a* - *b*) values for the heterogeneous faults. The actual (*a* - *b*) values from the heterogeneous experiments are higher than predicted, suggesting that the frictional properties of the clay patches contribute more to the average (*a* - *b*) values, as would be consistent with higher normal stresses in the clay patches than in the quartz patch due to differential compaction which could also potentially explain some of the progressive weakening trends in our experiments (see Supplementary Note 1 below for full discussion of this effect). Note, however, that even though the

experimental (*a* - *b*) values are higher than predicted for the heterogeneous faults, they are still consistently lower than (*a* - *b*) values from the equivalent homogeneous faults. Also note that only (*a* - *b*) values from up-steps in the sliding velocity (from 0.3 to 3 $\mu m \cdot s^{-1}$) are shown in the figure as there is asymmetry in the frictional response between velocity up-steps and down-steps (from 3 to 0.3 $\mu m \cdot s^{-1}$), something that has been reported in previous studies on the rate-dependent frictional behaviour of fault gouges[3–5].

**Heterogeneous faults**

| Displ: | 1.5 mm | | | 2.5 mm | | | 3.5 mm | | | 4.5 mm | | | 5.5 mm | | | 6.5 mm | | | 7.5 mm | | |
|---|---|---|---|---|---|---|---|---|---|---|---|---|---|---|---|---|---|---|---|---|---|
| Clay (%) | *a* ($10^{-3}$) | *b* ($10^{-3}$) | *Dc* (μm) | *a* ($10^{-3}$) | *b* ($10^{-3}$) | *Dc* (μm) | *a* ($10^{-3}$) | *b* ($10^{-3}$) | *Dc* (μm) | *a* ($10^{-3}$) | *b* ($10^{-3}$) | *Dc* (μm) | *a* ($10^{-3}$) | *b* ($10^{-3}$) | *Dc* (μm) | *a* ($10^{-3}$) | *b* ($10^{-3}$) | *Dc* (μm) | *a* ($10^{-3}$) | *b* ($10^{-3}$) | *Dc* (μm) |
| **0** | 3.1 | 5.1 | 27 | - | - | - | - | - | - | - | - | - | - | - | - | - | - | - | - | - | - |
| **20** | 1.9 | 2.8 | 43 | - | - | - | - | - | - | - | - | - | - | - | - | - | - | - | - | - | - |
| **30** | 3.1 | 4.4 | 61 | 10.5 | 9.1 | 8 | 2.9 | 1.7 | 61 | 9.8 | 7.5 | 4 | 3.3 | 0.4 | 21 | 7.0 | 4.7 | 6 | 2.8 | 2.2 | 79 |
| **40** | 2.4 | 2.8 | 32 | 1.8 | 0.4 | 272 | 2.5 | 1.0 | 22 | 3.7 | 0.6 | 85 | 2.6 | 0.5 | 39 | 1.9 | -1.3 | 53 | 2.6 | -0.2 | 103 |
| **50** | 4.0 | 2.7 | 26 | 12.2 | 10.4 | 3 | 4.3 | 1.6 | 44 | 3.2 | -0.8 | 57 | 4.2 | 1.5 | 69 | 3.9 | -1.3 | 156 | 3.9 | -0.8 | 112 |
| **60** | 4.4 | 1.8 | 19 | 3.4 | -0.5 | 271 | 4.9 | 1.8 | 45 | 3.4 | -1.3 | 166 | 0 | -4.6 | 10 | 5.5 | -0.5 | 186 | 0 | -4.3 | 6 |
| **70** | 5.7 | 2.0 | 65 | 3.2 | -0.5 | 45 | 4.6 | 0.9 | 17 | 4.1 | -1.4 | 106 | 4.4 | 0.3 | 12 | 5.3 | -0.8 | 74 | 4.8 | 1.6 | 29 |
| **80** | 4.3 | 2.3 | 28 | 5.5 | 1.0 | 107 | 6.7 | 1.6 | 14 | 6.0 | 0.4 | 17 | 5.6 | 1.3 | 11 | 5.2 | -0.6 | 99 | 5.0 | 0.4 | 122 |
| **100** | 6.8 | 1.9 | 22 | 6.7 | 0.8 | 120 | 6.7 | 2.0 | 21 | 7.0 | 0.6 | 210 | 6.6 | 2.2 | 40 | 6.8 | 0.4 | 136 | 6.8 | 0.8 | 28 |

**Homogeneous faults**

| Displ: | 1.5 mm | | | 2.5 mm | | | 3.5 mm | | | 4.5 mm | | | 5.5 mm | | | 6.5 mm | | | 7.5 mm | | |
|---|---|---|---|---|---|---|---|---|---|---|---|---|---|---|---|---|---|---|---|---|---|
| Clay (%) | *a* ($10^{-3}$) | *b* ($10^{-3}$) | *Dc* (μm) | *a* ($10^{-3}$) | *b* ($10^{-3}$) | *Dc* (μm) | *a* ($10^{-3}$) | *b* ($10^{-3}$) | *Dc* (μm) | *a* ($10^{-3}$) | *b* ($10^{-3}$) | *Dc* (μm) | *a* ($10^{-3}$) | *b* ($10^{-3}$) | *Dc* (μm) | *a* ($10^{-3}$) | *b* ($10^{-3}$) | *Dc* (μm) | *a* ($10^{-3}$) | *b* ($10^{-3}$) | *Dc* (μm) |
| **0** | 3.1 | 5.1 | 27 | - | - | - | - | - | - | - | - | - | - | - | - | - | - | - | - | - | - |
| **20** | 3.1 | 2.8 | 21 | 8.4 | 6.0 | 49 | 5.6 | 4.3 | 6 | 13.3 | 9.0 | 11 | 5.5 | 4.3 | 6 | 13.8 | 9.9 | 7 | 8.6 | 6.7 | 3 |
| **30** | 5.5 | 4.9 | 11 | 12.3 | 7.8 | 21 | 7.0 | 5.5 | 5 | 12.9 | 8.4 | 13 | 11.5 | 8.7 | 3 | 15.0 | 11.4 | 6 | 17.3 | 15.0 | 1 |
| **40** | 4.8 | 2.9 | 17 | 11.7 | 5.9 | 17 | 6.1 | 2.5 | 10 | 13.7 | 7.9 | 5 | 21.9 | 17.0 | 1 | 12.4 | 6.5 | 7 | 8.7 | 3.8 | 3 |
| **50** | 5.8 | 1.6 | 27 | 7.6 | 1.8 | 44 | 6.0 | 1.2 | 18 | 30.5 | 24.5 | 2 | 6.5 | 1.6 | 17 | 8.3 | 2.2 | 7 | 5.2 | 0.9 | 153 |
| **60** | 6.7 | 2.5 | 37 | 9.0 | 3.2 | 25 | 5.5 | 1.4 | 46 | 16.9 | 9.1 | 4 | 6.2 | 0.6 | 16 | 17.7 | 10.2 | 3 | 5.7 | 1.6 | 35 |
| **70** | 5.7 | 1.1 | 26 | 7.9 | 2.5 | 31 | 6.3 | 1.7 | 36 | 7.8 | 1.5 | 37 | 5.4 | 1.4 | 33 | 7.5 | 1.4 | 99 | 5.6 | 1.5 | 59 |
| **80** | 6.3 | 2.4 | 37 | 9.3 | 2.6 | 22 | 5.6 | 2.0 | 55 | 7.4 | 1.3 | 63 | 6.9 | 2.0 | 22 | 7.6 | 1.0 | 101 | 5.9 | 1.1 | 49 |
| **100** | 6.8 | 1.9 | 22 | 6.7 | 0.8 | 120 | 6.7 | 2.0 | 21 | 7.0 | 0.6 | 210 | 6.6 | 2.2 | 40 | 6.8 | 0.4 | 136 | 6.0 | 0.8 | 28 |

**Heterogeneous faults (reversed symmetry)**

| Displ: | 1.5 mm | | | 2.5 mm | | | 3.5 mm | | | 4.5 mm | | | 5.5 mm | | | 6.5 mm | | | 7.5 mm | | |
|---|---|---|---|---|---|---|---|---|---|---|---|---|---|---|---|---|---|---|---|---|---|
| Clay (%) | *a* ($10^{-3}$) | *b* ($10^{-3}$) | *Dc* (μm) | *a* ($10^{-3}$) | *b* ($10^{-3}$) | *Dc* (μm) | *a* ($10^{-3}$) | *b* ($10^{-3}$) | *Dc* (μm) | *a* ($10^{-3}$) | *b* ($10^{-3}$) | *Dc* (μm) | *a* ($10^{-3}$) | *b* ($10^{-3}$) | *Dc* (μm) | *a* ($10^{-3}$) | *b* ($10^{-3}$) | *Dc* (μm) | *a* ($10^{-3}$) | *b* ($10^{-3}$) | *Dc* (μm) |
| **40** | 4.0 | 3.8 | 31 | 12.1 | 10.5 | 3 | 5.5 | 2.7 | 28 | 4.1 | 1.2 | 150 | 6.1 | 3.1 | 47 | 5.4 | 0.7 | 98 | - | - | - |
| **60** | 4.8 | 2.0 | 26 | 3.6 | 0.2 | 9 | 5.5 | 1.9 | 38 | 3.9 | -0.7 | 121 | 5.0 | 0.3 | 8 | 6.1 | -0.4 | 152 | 4.9 | 0.4 | 10 |

**Supplementary Table 1| Processed values of the rate-and-state parameters *a, b* and *Dc* from all the velocity steps in this study.** Data are shown for both heterogeneous and homogeneous faults with varying clay-quartz compositions (displayed as % clay fraction). There are a total of seven velocity steps for each

experiment (every 1 mm of displacement, starting at a displacement of 1.5 mm). Note that for displacements >1.5 mm, the rate-and-state parameters could not be calculated for the endmember quartz fault (i.e. 0% clay fraction) or the heterogeneous fault with a 20% clay fraction, as stick-slip instabilities were triggered by the velocity steps; these values have therefore been left blank. Also shown are the processed *a*, *b* and *Dc* values from the heterogeneous fault experiments with a reversed symmetry, where a central clay patch is bound by two quartz patches (Supplementary Fig. 3).

**Supplementary Note 1**

*Estimating the potential effect of differential compaction on interface weakening*

The normal stress applied to the sample by the confining pressure ($P_c$) and the pore fluid pressure ($P_f$) within the gouge are held constant during all tests in this study, resulting in the constant average effective normal stress $\overline{\sigma_n} = P_c - P_f$. So there should be no evolving elastic compaction during the tests, and any inferred compaction is inelastic. Both the quartz and clay gouge layers have the same thickness at each moment of time in the experiment, constrained by the steel assembly that is effectively rigid compared to the elastic properties of the gouge. Assuming that the total normal stress is the sum of the elastic and inelastic parts, any differential inelastic compression between the quartz and clay gouge would result in different elastic fault-normal strain in the two gouges and hence potentially different normal stresses, causing redistribution of normal stress. For this redistribution to result in a progressive weakening of shear resistance, the normal stress has to increase on the weaker, clay phase, which would require that clay compacts less (and quartz compacts more). This is indeed the case (Supplementary Fig. 2).

To compensate for the larger compression of the quartz gouge during the loading phase, extra thickness of the quartz gouge material was initially applied. This procedure worked, since the peak value of the friction coefficient at the end of the loading phase, where the friction curve turns the corner into frictional sliding, for all heterogeneous fault experiments is consistent with both clay and gouge layers being at the same applied effective normal stress. For example, for the heterogeneous interface with the clay fraction $f_{cl}$ of 0.4 (or 40% of the interface filled with clay gouge), the peak friction coefficient is about 0.5, which is quite similar to the sum of the contributions from both quartz and clay layers at the same normal stress, $\mu_{cl} f_{cl} + \mu_{qu}(1 - f_{cl}) = 0.27 \cdot 0.4 + 0.7 \cdot 0.6 = 0.52$, where $\mu_{cl} = 0.27$ and $\mu_{qu} = 0.7$ are the near-constant friction coefficients of the clay and quartz gouges, respectively, from the homogeneous interface tests. Hence it appears that the quartz and clay gouge layers are at about the same effective normal stress at the end of the loading phase, as intended.

However, there is much smaller but continuing differential compaction between quartz and clay in the frictional sliding regime (Stage II of Supplementary Fig. 2 which starts at about 1 mm of displacement), of about 20 μm more compaction in quartz, based on the homogenous interface tests. This corresponds to normal strain of about 0.02 for the (rounded) layer thickness of 1 mm. The following highly simplified calculation allows us to estimate the potential impact of such differential compaction on the average friction coefficient. The additional fault-normal compressive elastic strain $\epsilon^{add}$ of 0.02 in the clay layer (which would arise due to the same thickness of both quartz and clay layers) would correspond to the additional fault normal compressive stress of $\sigma^{add} = \left(K + \frac{4G}{3}\right)\epsilon^{add}$, where $K$ and $G$ are the bulk and shear modulus of the clay layer, respectively. Assuming $\left(K + \frac{4G}{3}\right)$ of 2 to 4 GPa (ref. 6), we get $\sigma^{add}$ in the clay gouge of 40 to 80 MPa, which implies significant redistribution of normal stress. Since the normal stresses in the clay and quartz gouges have to balance the externally applied normal stress, we can find the normal stresses $\sigma_{qu}$ and $\sigma_{cl} = \sigma_{qu} + \sigma^{add}$ in the quartz and clay gouges, respectively, from $\sigma_{cl}f_{cl} + \sigma_{qu}(1 - f_{cl}) = P_c$. For example, for the clay fraction $f_{cl} = 0.4$, $P_c$ = 60 MPa, and $\sigma^{add} = 40$ MPa, one gets $\sigma_{qu} = 44$ MPa and $\sigma_{cl} = \sigma_{qu} + \sigma^{add} = 84$ MPa, a substantial difference between the normal stress in the two layers. Since the weaker clay gouge would support more of the normal load, the interface would become weaker than for the spatially uniform normal stress. The combined friction coefficient $\mu^{comb}$ of the two layers with such normal stress redistribution due to differential compaction would then be given by $\mu^{comb} = [(\sigma_{cl} - P_f)\mu_{cl}f_{cl} + (\sigma_{qu} - P_f)\mu_{qu}(1 - f_{cl})]/(P_c - P_f) = 0.43$ for $f_{cl} = 0.4$ and $P_f = 20$ MPa. The resulting average interface friction coefficient of $\mu^{comb} = 0.43$ is substantially weaker than the peak one of 0.5, accounting for about half of the observed friction reduction from 0.5 to 0.35 for the 40% clay case of Fig. 1c. If this calculation is redone with the (higher) additional compressive stress of 80 MPa (corresponding to higher elastic moduli of clay gouge), one gets $\mu^{comb} = 0.32$, even slightly more than the observed reduction.

We conclude that the differential compaction between the quartz and clay gouges during shear - and the associated normal stress redistribution - may account for a significant portion of the observed interface weakening, with some caveats that would require additional study. For example, the compaction data of Supplementary Fig. 2 is obtained under the constant effective normal stress, whereas the redistribution of normal stress due to differential compaction in heterogeneous samples would result in varying normal stress for both quartz and clay layers, potentially resulting in different evolution of compaction. Furthermore, the elastic properties of the gouge layers are not precisely known and may evolve with compaction and changing normal stress. At the same time, such differential compaction may be important during shear of heterogeneous natural faults and requires further experimental and theoretical study.

**Supplementary References**


1. Faulkner, D. R., Sanchez-Roa, C., Boulton, C. & den Hartog, S. A. M. Pore fluid pressure development in compacting fault gouge in theory, experiments, and nature. *J. Geophys. Res. Solid Earth* **123**, 226–241 (2018).

2. Crawford, B. R., Faulkner, D. R. & Rutter, E. H. Strength, porosity, and permeability development during hydrostatic and shear loading of synthetic quartz-clay fault gouge. *J. Geophys. Res.* **113**, (2008).

3. Rathbun, A. P. & Marone, C. Symmetry and the critical slip distance in rate and state friction laws. *J. Geophys. Res. Solid Earth* **118**, 3728–3741 (2013).

4. Bedford, J. D., Faulkner, D. R., Allen, M. J. & Hirose, T. The stabilizing effect of high pore-fluid pressure along subduction megathrust faults: Evidence from friction experiments on accretionary sediments from the Nankai Trough. *Earth Planet. Sci. Lett.* **574**, 117161 (2021).

5. Xing, T., Zhu, W., French, M. & Belzer, B. Stabilizing effect of high pore fluid pressure on slip

behaviors of gouge-bearing faults. *J. Geophys. Res. Solid Earth* **124**, 9526–9545 (2019).

6. Knuth, M. W., Tobin, H. J. & Marone, C. Evolution of ultrasonic velocity and dynamic elastic moduli with shear strain in granular layers. *Granul. Matter* **15**, 499–515 (2013).
